# How and under what circumstances do quality improvement collaboratives lead to better outcomes? A systematic review

**DOI:** 10.1186/s13012-020-0978-z

**Published:** 2020-05-04

**Authors:** Karen Zamboni, Ulrika Baker, Mukta Tyagi, Joanna Schellenberg, Zelee Hill, Claudia Hanson

**Affiliations:** 1 Department of Disease Control, London School of Hygiene and Tropical Medicine, Keppel Street, London, WC1E 7HT UK; 2 Department of Public Health Sciences, Karolinska Institutet, Stockholm, Sweden; 3 Department of Family Medicine, College of Medicine, University of Malawi, Blantyre, Malawi; 4 Public Health Foundation, Kavuri Hills, Madhapur, Hyderabad, India; 5 Institute for Global Health, University College London, London, UK

**Keywords:** Quality improvement, Evaluation, Realist synthesis, Context, Mechanism of change

## Abstract

**Background:**

Quality improvement collaboratives are widely used to improve health care in both high-income and low and middle-income settings. Teams from multiple health facilities share learning on a given topic and apply a structured cycle of change testing. Previous systematic reviews reported positive effects on target outcomes, but the role of context and mechanism of change is underexplored. This realist-inspired systematic review aims to analyse contextual factors influencing intended outcomes and to identify how quality improvement collaboratives may result in improved adherence to evidence-based practices.

**Methods:**

We built an initial conceptual framework to drive our enquiry, focusing on three context domains: health facility setting; project-specific factors; wider organisational and external factors; and two further domains pertaining to mechanisms: intra-organisational and inter-organisational changes. We systematically searched five databases and grey literature for publications relating to quality improvement collaboratives in a healthcare setting and containing data on context or mechanisms. We analysed and reported findings thematically and refined the programme theory.

**Results:**

We screened 962 abstracts of which 88 met the inclusion criteria, and we retained 32 for analysis. Adequacy and appropriateness of external support, functionality of quality improvement teams, leadership characteristics and alignment with national systems and priorities may influence outcomes of quality improvement collaboratives, but the strength and quality of the evidence is weak. Participation in quality improvement collaborative activities may improve health professionals’ knowledge, problem-solving skills and attitude; teamwork; shared leadership and habits for improvement. Interaction across quality improvement teams may generate normative pressure and opportunities for capacity building and peer recognition.

**Conclusion:**

Our review offers a novel programme theory to unpack the complexity of quality improvement collaboratives by exploring the relationship between context, mechanisms and outcomes. There remains a need for greater use of behaviour change and organisational psychology theory to improve design, adaptation and evaluation of the collaborative quality improvement approach and to test its effectiveness. Further research is needed to determine whether certain contextual factors related to capacity should be a precondition to the quality improvement collaborative approach and to test the emerging programme theory using rigorous research designs.

Contribution to the literature

- Quality improvement collaboratives are a widely used approach. However, solid evidence of their effectiveness is limited and research suggests that achievement of results is highly contextual.
- Previous research on the role of context in quality improvement collaboratives has not explored the dynamic relationship between context, mechanisms and outcomes. We systematically explore these through a review of peer-reviewed and grey literature.
- Understanding contextual factors influencing intended quality improvement collaborative outcomes and the mechanisms of change can aid implementation design and evaluation. This systematic review offers a novel programme theory to unpack the complexity of quality improvement collaboratives.

## Background

Improving quality of care is essential to achieve Universal Health Coverage [1]. One strategy for quality improvement is quality improvement collaboratives (QIC) defined by the Breakthrough Collaborative approach [2]. This entails teams from multiple health facilities working together to improve performance on a given topic supported by experts who share evidence on best practices. Over a short period, usually 9–18 months, quality improvement coaches support teams to use rapid cycle tests of change to achieve a given improvement aim. Teams also attend “learning sessions” to share improvement ideas, experience and data on performance [2–4]. Collaboration between teams is assumed to shorten the time required for teams to diagnose a problem and identify a solution and to provide an external stimulus for innovation [2, 3].

QICs are widely used in high-income countries and proliferating in low- and middle-income countries (LMICs), although solid evidence of their effectiveness is limited [5–11]. A systematic review on the effects of QICs, largely focused on high-income settings, found that three quarters of studies reported improvement in at least half of the primary outcomes [7]. A previous review suggested that evidence on QICs effectiveness is positive but highly contextual [5], and a review of the effects of QICs in LMICs reported a positive and sustained effect on most indicators [12]. However, there are important limitations. First, with one exception [11], systematic reviews define QIC effectiveness on the basis of statistically significant improvement in at least one, or at least half of “primary” outcomes [7, 12] neglecting the heterogeneity of outcomes and the magnitude of change. Second, studies included in the reviews are weak, most commonly before-after designs, while most randomised studies give insufficient detail of randomisation and concealment procedures [7], thus potentially overestimating the effects [13]. Third, most studies use self-reported clinical data, introducing reporting bias [8–10]. Fourth, studies generally draw conclusions based on facilities that completed the programme, introducing selection bias. Recent well-designed studies support a cautious assessment of QIC effectiveness: a stepped wedge randomised controlled trial of a QIC intervention aimed at reducing mortality after abdominal surgery in the UK found no evidence of a benefit on survival [14]. The most robust systematic review of QICs to date reports little effect on patient health outcomes (median effect size (MES) less than 2 percentage points), large variability in effect sizes for different types of outcomes, and a much larger effect if QICs are combined with training (MES 111.6 percentage points for patient health outcomes; and MES of 52.4 to 63.4 percentage points for health worker practice outcomes) [11]. A review of group problem-solving including QIC strategies to improve healthcare provider performance in LMICs, although mainly based on low-quality studies, suggested that these may be more effective in moderate-resource than in low-resource settings and their effect smaller with higher baseline performance levels [6].

Critiques of quality improvement suggest that the mixed results can be partly explained by a tendency to reproduce QIC activities without attempting to modify the functioning, interactions or culture in a clinical team, thus overlooking the mechanisms of change [15]. QIC implementation reports generally do not discuss how changes were achieved, and lack explicit assumptions on what contextual factors would enable them; the primary rationale for using a QIC often being that it has been used successfully elsewhere [7] . In view of the global interest in QICs, better understanding of the influence of context and of mechanisms of change is needed to conceptualise and improve QIC design and evaluation [6, 7]. In relation to context, a previous systematic review explored determinants of QIC success, reporting whether an association was found between any single contextual factor and any effect parameter. The evidence was inconclusive, and the review lacked an explanatory framework on the role of context for QIC success [16]. Mechanisms have been documented in single case studies [17] but not systematically reviewed.

In this review, we aim to analyse contextual factors influencing intended outcomes and to identify how quality improvement collaboratives may result in improved adherence to evidence-based practices, i.e. the mechanisms of change.

## Methods

**Fig. 1:**
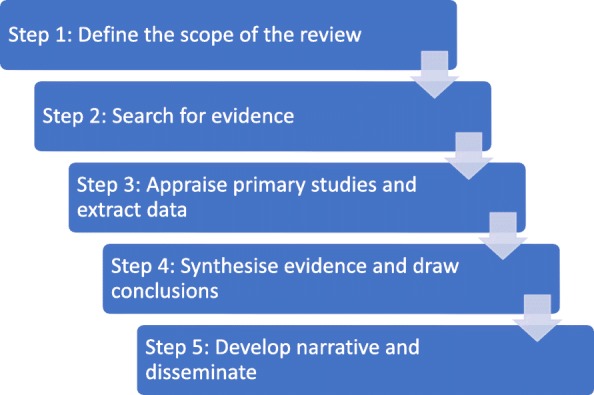
Realist review process, adapted from Pawson R. et al. 2015 [18]

This review is inspired by the realist review approach, which enables researchers to explore how, why and in what contexts complex interventions may work (or not) by focusing on the relationships between context, mechanisms and outcomes [18–20]. The realist review process consists of 5 methodological steps (Fig. 1). We broadly follow this methodological guidance with some important points of departure from it. We had limited expert engagement in developing our theory of change, and our preliminary conceptual framework was conceived as a programme theory [21] rather than as a set of context-mechanism-outcomes configurations (step 1) [22]. We followed a systematic search strategy driven by the intervention definition with few iterative searches [19], and we included a quality appraisal of the literature because the body of evidence on our questions is generally limited by self-reporting of outcomes, selection and publication bias [7, 9, 15].

### Clarifying scope of the review

**Fig. 2:**
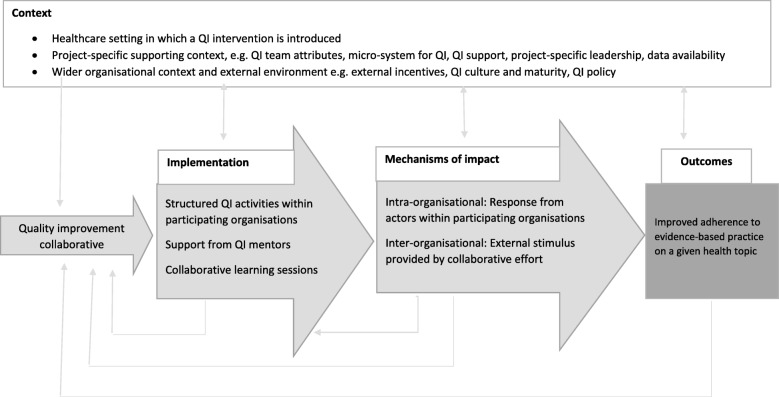
Review conceptual framework (adapted from MRC process evaluation framework)

We built an initial conceptual framework to drive our enquiry (Fig. 2) in the form of a preliminary programme theory [21, 23]. We adapted the Medical Research Council process evaluation framework [24] using findings from previous studies [8, 16, 25, 26] to conceptualise relationships between contextual factors, mechanisms of change and outcomes. We defined context as “factors external to the intervention which may influence its implementation” [24].We drew from Kaplan’s framework to understand context for quality improvement (MUSIQ), which is widely used in high-income countries, and shows promise for LMIC settings [27, 28]. We identified three domains for analysis: the healthcare setting in which a quality improvement intervention is introduced; the project-specific context, e.g. characteristics of quality improvement teams, leadership in the implementing unit, nature of external support; and the wider organisational context and external environment [29].

We defined mechanisms of change as the “underlying entities, processes, or structures which operate in particular contexts to generate outcomes of interest” [30]. Our definition implies that mechanisms are distinct from, but linked to, intervention activities: intervention activities are a *resource* offered by the programme to which participants respond through cognitive, emotional or organisational processes, influenced by contextual factors [31]. We conceptualised the collaborative approach as a structured *intervention* or *resource* to embed innovative practices into healthcare organisations and accelerate diffusion of innovations based on seminal publications on QICs [2, 3]. Strategies described in relation to implementation of a change, e.g. “making a change the normal way” that an activity is done [3], implicitly relate to normalisation process theory [17, 32] . Spreading improvement is explicitly inspired by the diffusion of innovation theory, attributing to early adopters the role of assessing and adapting innovations to facilitate their spread, and the role of champions for innovation, exercising positive peer pressure in the collaborative [3, 17, 33]. Therefore, we identified two domains for analysis of mechanisms of change: we postulated that QIC outcomes may be generated by mechanisms activated within each organisation (intra-organisational mechanisms) and through their collaboration (inter-organisational mechanisms). When we refer to QIC outcomes, we refer to measures which an intervention aimed to influence, including measures of clinical processes, perceptions of care, patient recovery, or other quality measures, e.g. self-reported patient safety climate.

KZ and JS discussed the initial programme theory with two quality improvement experts acknowledged at the end of this paper. They suggested alignment with the MUSIQ framework and commented on the research questions, which were as follows:

Context

1. In what kind of health facility settings may QICs work (or not)? (focus on characteristics of the health facility setting)
2. What defines an enabling environment for QICs? (focus on proximate project-specific factors and on wider organisational context and external environment)

Mechanisms

3. How may engagement in QICs influence health workers and the organisational context to promote better adherence to evidence-based guidelines? (focus on intra-organisational mechanisms)
4. What is it about collaboration with other facilities that may lead to better outcomes? (focus on inter-organisational mechanisms)

### Search strategy

**Fig. 3:**
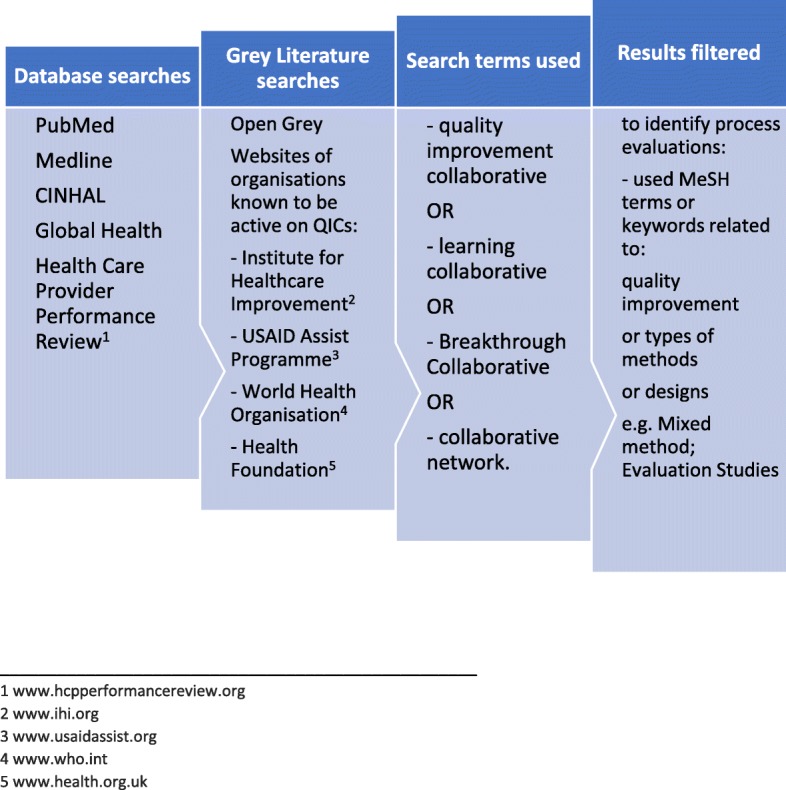
Search strategy

The search strategy is outlined in Fig. 3 and detailed in Additional file 1. Studies were included if they (i) referred to the quality improvement collaborative approach [2, 5, 8, 16], defined in line with previous reviews as consisting of all the following elements: a specified topic; clinical and quality improvement experts working together; multi-professional quality improvement teams in multiple sites; using multiple rapid tests of change; and a series of structured collaborative activities in a given timeframe involving learning sessions and visits from mentors or facilitators (ii) were published in English, French or Spanish, from 1997 to June 2018; and (iii) referred to a health facility setting, as opposed to community, administrative or educational setting.

Studies were excluded if they focused on a chronic condition, palliative care, or administrative topics, and if they did not contain primary quantitative or qualitative data on process of implementation, i.e. the search excluded systematic reviews; protocol papers, editorials, commentaries, methodological papers and studies reporting exclusively outcomes of QIC collaboratives or exclusively describing implementation without consideration of context or mechanisms of change.

### Screening

We applied inclusion and exclusion criteria to titles and abstracts and subsequently to the full text. We identified additional studies through references of included publications and backward and forward citation tracking.

### Data collection

We developed and piloted data extraction forms in MS Excel. We classified studies based on whether they focused on context or mechanisms of change and captured qualitative and quantitative data under each component. Data extraction also captured the interaction between implementation, context and mechanisms, anticipating that factors may not fit neatly into single categories [18, 19].

KZ and MT independently conducted a structured quality appraisal process using the STROBE checklist for quantitative observational studies, the Critical Appraisal Skills Programme checklist for qualitative studies and the Mixed Methods Appraisal Tool for mixed method studies [34–37] and resolving disagreement by consensus. To aid comparability, given the heterogeneity of study designs, a score of 1 was assigned to each item in the checklist, and a total score was calculated for each paper. Quality was rated low, medium or high for papers scoring in the bottom half, between 50 and 80%, or above 80% of the maximum score. We did not exclude studies because of low quality: in all such cases, both authors agreed on the study’s relative contribution to the research questions [19, 38].

### Synthesis and reporting of results

Analysis was informed by the preliminary conceptual framework (Fig. 2) and conducted thematically by framework domain by the lead author. We clustered studies into context and mechanism. Under context, we first analysed quantitative data to identify factors related to the framework and evidence of their associations with mechanisms and outcomes. Then, from the qualitative evidence, we extracted supportive or dissonant data on the same factors. Under mechanisms, we identified themes under the two framework domains using thematic analysis. We generated a preliminary coding framework for context and mechanism data in MS Excel. UB reviewed a third of included studies, drawn randomly from the list stratified by study design, and independently coded data following the same process. Disagreements were resolved through discussion. We developed a final coding framework, which formed the basis of our narrative synthesis of qualitative and quantitative data.

We followed the RAMESES reporting checklist, which is modelled on the PRISMA statement [39] and tailored for reviews aiming to highlight relationships between context, mechanisms and outcomes [40] (Additional file 2). All included studies reported having received ethical clearance.

## Results

### Search results

**Fig. 4:**
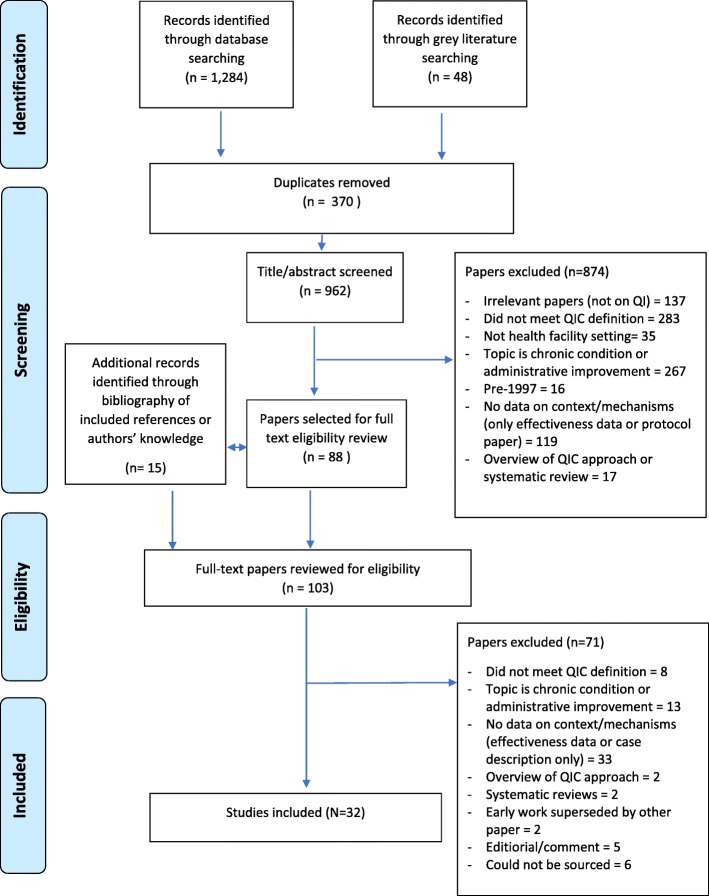
Search flowchart

Searches generated 1,332 results. After removal of duplicates (370), 962 abstracts were screened of which 88 met the inclusion criteria. During the eligibility review process, we identified 15 papers through bibliographies of eligible papers and authors’ suggestions. Of the 103 papers reviewed in full, 32 met inclusion criteria and were retained for analysis (Table 1). Figure 4 summarises the search results.

**Table 1:** Overview of included studies

No.	Author (ref)	Year	Country	Collaborative name	Topic	Study aim	Health setting	No. facilities (individuals) in study	Study design	Published	Focus
1	Amarasingham et al.	2007	USA	Keystone Intensive Care Units Project	Central line associated bloodstream infection	Assess correlation between automation and usability of clinical information systems and clinical outcomes.	Intensive care unit	19 (19)	Uncontrolled before-after	Peer-reviewed	Context
2	Ament et al.	2014	Netherlands	ERAS (Enhanced Recovery after surgery)	Colonic surgery	Explore strategies for sustaining ERAS	Hospitals	10 (18)	Qualitative	Peer-reviewed	Mechanism
3	Baker et al.	2018	Tanzania	EQUIP (Expanded Quality Management using Information Power)	Maternal and newborn health	Investigate how different components of a QIC were understood and experienced by health workers, and contributed to its mechanisms of effect	District hospital, health centre and dispensaries	13(16)	Qualitative	Peer-reviewed	Mechanism
4	Benn et al.	2009	UK	Safer Patient Initiative	Patient safety	Understand participants’ perception of impact of the pilot programme	NHS Health Trusts	4	Mixed methods: cross-sectional and qualitative	Peer-reviewed	Mechanism and implementation
5	Benn et al.	2012	UK	Safer Patient Initiative	Patient safety	Analyse impact of intervention of safety culture and climate and role of contextual and programme factors in changes.	NHS Health Trusts	19 [2 merged in 1] (284)	Uncontrolled before-after	Peer-reviewed	Context and implementation
6	Burnett et al.	2009	UK	Safer Patient Initiative	Patient safety	Analyse perceptions of organisational readiness and its relationship with intervention impact	NHS Health Trusts	4 (41)	Mixed methods: cross-sectional and qualitative	Peer-reviewed	Context
7	Carlhed et al.	2006	Sweden	Quality Improvement in Coronary Care	Acute myocardial infarction (AMI)	Evaluate effect of QIC on adherence to AMI guidelines	Hospitals	19 + 19 controls	Non-randomised controlled before and after	Peer-reviewed	Context
8	Carter et al.	2014	UK	Stroke 90:10	Stroke	Explain processes and outcomes of the QIC intervention	Hospitals	11(32)	Qualitative	Peer-reviewed	Mechanism
9	Colbourn et al.	2013	Malawi	MaiKhanda	Maternal and newborn health	Evaluate impact and processes of change	Hospitals and health centres	9 and 29	Mixed methods: cross-sectional and qualitative	Grey	Context, mechanism and implementation
10	Catsambas et al.	2008	LMICs various	35 collaboratives funded by USAID between 2002 - 2007	Various: Maternal and newborn health, nutrition, HIV/AIDS	Document and evaluate the implementation and results of the Quality Assurance Project	Hospitals and health centres	N/A	External review - multiple projects	Grey	Context, mechanism & implementation
11	Dainty et al.	2013	Canada	Ontario Intensive Care Units Best Practice Project	Evidence-based care practices in Intensive Care Units	Understand staff perspectives on QIC and hypothesise theoretical constructs that might explain the effect of collaboration	Hospitals	12 (32)	Qualitative	Peer-reviewed	Mechanism
12	Dixon-Woods et al.	2011	USA	Keystone ICU Project	Central line associated bloodstream infection	Develop an ex-post theory of the project	Intensive Care Units	n/a	Case description	Peer-reviewed	Mechanism
13	Duckers et al.	2009	Netherlands	Better Faster	Patient safety	Test whether consensus on perceived leadership support among physicians influences the relation between physician’s perception and participation.	Hospitals	8 (864)	Cross-sectional	Peer-reviewed	Context
14	Duckers M. et al.	2009	Netherlands	Better Faster	Patient safety	Assess relations between conditions for successful implementation, applied changes, perceived success and actual outcomes.	Hospitals	23 (237)	Cross-sectional	Peer-reviewed	Context, mechanism and implementation
15	Duckers M. et al.	2011	Netherlands	Better Faster	Patient safety	Describe how the first group of hospitals sustained and disseminated improvements	Hospitals	8 (8)	Qualitative	Peer-reviewed	Mechanism
16	Duckers M. et al.	2014	Netherlands	Better Faster	Patient safety	Test whether perceived average project success at QIC level explains dissemination of projects.	Hospitals	16 (84 out of 148)	Cross-sectional	Peer-reviewed	Mechanism
17	Feldman-Winter et al.	2016	USA	Best Fed Beginnings	Breastfeeding	Describe collaborative and present lessons learned from implementation.	Hospitals	89(89)	Case description	Peer-reviewed	Mechanism and implementation
18	Horbar et al.	2003	USA	Vermont Oxford Network Newborn Intensive Care Units /Q 2000	Quality and safety of neonatal intensive care	Describe collaborative and present implementation strategy.	Hospitals		Case description	Peer-reviewed	Context, mechanism and implementation
19	Jaribu et al.	2016	Tanzania	INSIST	Maternal and newborn health	Describe health workers’ perceptions of a QIC intervention	Health centres and dispensaries	11 (15)	Qualitative	Peer-reviewed	Mechanism
20	Linnander et al.	2016	Ethiopia	Ethiopian Hospital Alliance for Quality	Patient satisfaction with hospital care	Analyse impact of QIC	Hospitals	68	Cross-sectional and uncontrolled before - after	Peer-reviewed	Context and implementation
21	Marquez et al.	2014	38 LMICs	Health Care Improvement Project	various	Document and evaluate the implementation and results of the Health Care Improvement project	various	N/A	External review - multiple projects	Grey	Context, mechanism and implementation
22	McInnes et al.	2007	USA	HIV collaborative under HRSA/HAB	HIV/AIDS	Assess whether participation in QIC changes care processes, systems and organisation of outpatient HIV clinics	HIV clinics	52 (104) Intervention and 35 (90) Controls from non QIC sites.	Non-randomised controlled before and after	Peer-reviewed	Context
23	Mills and Weeks	2004	USA	5 Veteran Health Association collaboratives between 1999 - 2001	Various	To identify the organisational, interpersonal and systemic characteristics of successful improvement teams	Hospitals	134 medical QITs in 5 BTS collaboratives	Uncontrolled before – after	Peer-reviewed	Context and implementation
24	Nembhard	2008	USA	4 collaboratives supported by IHI	Efficiency in primary care; complications in ICUs; reducing adverse drug events; reducing surgical site infections	Understand participants’ views of the relative helpfulness of various features of QICs	Hospitals	53 teams (217)	Mixed methods: cross-sectional and qualitative	Peer-reviewed	Mechanism
25	Nembhard	2012	USA	4 collaboratives supported by IHI	as above	Study the use of interorganizational learning activities as an explanation of mixed performance among collaborative participants	Hospitals	52 teams (48 hospitals)	Cross-sectional	Peer-reviewed	Mechanism
26	Osibo et al.	2017	Nigeria	Lafiyan Jikin Mata	HIV/AIDS	Discuss lessons learned from QIC implementation and analyse effect of QIC activities on process indicators.	Hospitals and PHC centres	32 (16 intervention + 16 controls)	Mixed methods: UBA and qualitative	Peer-reviewed	Mechanism and implementation
27	Parand et al.	2012	UK	Safer Patient Initiative	Patient safety	Identify strategies to facilitate the sustainability of the QIC	NHS Health Trusts	20 (35)	Qualitative	Peer-reviewed	Mechanism and implementation
28	Pinto et al.	2011	UK	Safer Patient Initiative	Patient safety	Evaluate influence of various factors on the perceived impact of QIC	NHS Health Trusts	20 (635)	Cross-sectional	Peer-reviewed	Mechanism
29	Rahimzai et al.	2014	Afghanistan	Maternal and Newborn Health Facility Demonstration Improvement Collaborative	Maternal and newborn health	Document implementation and describe results of a QIC project	Provincial hospitals, health centres and posts in provinces + large referral hospitals in Kabul	Participating facilities in “Demonstration wave”: 25 in provinces and 6 in Kabul: Wave 1–2: additional 6 facilities.	Case description	Peer-reviewed	Mechanism and implementation
30	Schouten et al.	2008	Netherlands	Stroke Collaborative I	Stroke	Explore effects of QIC and determinants of success	Stroke services	23	Cross-sectional and before - after with reference group	Peer-reviewed	Context
31	Sodzi-Tettey et al.	2013	Ghana	Project Fives Alive!	Maternal and newborn health	Document implementation, describe results and lessons learned of a QIC project	Hospitals (district and regional) and health centres	N/A	Case description	Grey	Context, mechanism and implementation
32	Stone et al.	2016	USA	California Perinatal Quality Care Collaborative	Breastfeeding	Assess factors that that affect sustained improvement following participation.	NICUs	6 (n/s)	Qualitative	Peer-reviewed	Mechanism

### Characteristics of included studies

Included studies comprised QIC process evaluations using quantitative, qualitative, and mixed methods designs, as well as case descriptions in the form of programme reviews by implementers or external evaluators, termed internal and independent programme reviews, respectively. While the application of QIC has grown in LMICs, evidence remains dominated by experiences from high-income settings: only 9 out of 32 studies were from a LMIC setting of which 4 were in the grey literature (Table 2).

**Table 2:** Overview of study focus, by country setting and study type

Focus	Total	Country setting		Internal or independent programme review	Before and after (controlled or uncontrolled)	Qualitative	Cross-sectional	Mixed methods
		High-income	Low or middle income					
Mechanism	12	10	2	1	0	7	3	1
Context	6	6	0	0	3	0	2	1
Context and implementation	3	2	1	0	2	0	1	0
Implementation and mechanism	5	3	2	2	0	1	0	2
All	6	2	4	4	0	0	1	1
Total	**32**	**23**	**9**	**7**	**5**	**8**	**7**	**5**

Most papers focused on mechanisms of change, either as a sole focus (38%) or in combination with implementation or contextual factors (72%) and were explored mostly through qualitative studies or programme reviews. The relative paucity of evidence on the role of context in relation to QIC reflects the gaps identified by other systematic reviews [7]. We identified 15 studies containing data on context of which 8 quantitatively tested the association between a single contextual factor and outcomes. Most studies were rated as medium quality (53%) with low ratings attributed to all internal and external programme reviews (Additional file 3). However, these were retained for analysis because of their rich accounts on the relationship between context, mechanisms and outcomes and the relative scarcity of higher quality evaluations taking into account this complexity [41].

### Context

We present results by research question in line with the conceptual framework (Fig. 2). We identified two research questions to explore three types of contextual factors (Table 3).

**Table 3:** Contextual factors

Category	No. of studies	Evidence synthesis				Quality of evidence (ref.)	
		Relationship with outcome		Relationship with mechanism		Quantitative and mixed methods	Qualitative and review
1 Healthcare setting in which a QI intervention is introduced							
Facility size	*N* = 1	No	No evidence that hospital size is associated with improvement in outcome.	-	Not discussed.	Medium [42]	
Base line performance	*N* = 1	Yes	Lower base line performance of hospitals is positively associated with magnitude of outcome improvement.	Yes	Lower base line performance is positively associated with active participation in QIC.	Medium [43]	
Voluntary or compulsory participation	*N* = 1	No	No evidence of differences in outcomes.	-	Not discussed.	High [44]	
Factors related to health facility readiness	*N* = 5	Yes/No	Inconclusive evidence of association between programme pre-conditions (staff, resources, usability of health information system systems, measurement data availability and senior level commitment to target) and outcomes.	Yes	Bottom up leadership style may foster more positive perceptions of organisational readiness for change. Limited clinical skills, poor staff morale and few resources negatively associated with outcomes.	Medium [42, 45, 46]; high [47]	Low [48]
2 Project-specific contextual factors							
External support	*N* = 6	Yes	Quality, appropriateness and intensity of quality improvement support positively associated with *perceived* improvement in outcomes.	Yes	The number of ideas tested by quality improvement teams partly mediates the association between external support and *perceived* improvement.	Medium [42, 46]; high [49]	Low [48, 50, 51]
Quality improvement team characteristics	*N* = 4	Yes	Inclusion of opinion leader, team functionality and previous knowledge or experience of quality improvement is positively associated with outcome.	-	Not discussed	Medium [52, 53]; high [49, 54]	
3 Wider organisational context and external environment							
Leadership characteristics	*N* = 5	Yes	Supportive leadership is positively associated with perceived improvement in outcomes.	Yes	Supportive leadership may motivate physicians to implement quality improvement and may enable active testing of ideas by quality improvement teams. Lack of supportive leadership may demotivate and stall quality improvement team efforts.	High [49, 54, 55]	Low [51, 56]
Health system alignment	*N* = 4	-	Not discussed.	Yes	Alignment with national priorities, national-level quality strategy, and incentives systems is essential to enable leadership support.	Medium [46]	Low [48, 50, 51]

#### In what kind of facility setting may QICs work (or not)?

The literature explored four healthcare setting characteristics: facility size, voluntary or compulsory participation in the QIC programme, baseline performance and factors related to health facility readiness. We found no conclusive evidence that facility size [42], voluntary or compulsory participation in the QIC programme [44], and baseline performance influence QIC outcomes [43]. For each of these aspects, we identified only one study, and those identified were not designed to demonstrate causality and lacked a pre-specified hypothesis on why the contextual factors studied would influence outcomes. As for heath facility readiness, this encompassed multiple factors perceived as programme preconditions, such as health information systems [42, 45, 47], human resources [42, 45, 46, 48] and senior level commitment to the target [42, 45]. There was inconclusive evidence on the relationships between these factors and QIC outcomes: the studies exploring this association quantitatively had mixed results and generally explored one factor each. A composite organisational readiness construct, combining the above-mentioned programme preconditions, was investigated in two cross-sectional studies from the same collaborative in a high-income setting. No evidence of an association with patient safety climate and capability was found, but this may have been due to limitations of the statistical model or of data collection on the composite construct and outcome measures [42, 45]. However, qualitative evidence from programme reviews and mixed-methods process evaluations of QIC programmes suggests that negative perceptions of the adequacy of available resources, low staff morale and limited availability of relevant clinical skills may contribute to negative perceptions of organisational readiness, particularly in LMIC settings. High-intensity support and partnership with other programmes may be necessary to fill clinical knowledge gaps [46, 48]. Bottom-up leadership may foster positive perceptions of organisational readiness for quality improvement [42, 46, 48].

#### What defines an enabling environment for QICs?

This question explored two categories in our conceptual framework: project-specific and wider organisational contextual factors. Project-specific contextual factors relate to the immediate unit in which a QIC intervention is introduced, and the characteristics of the QIC intervention that may influence its implementation [29]. We found mixed evidence that adequacy and appropriateness of external support for QIC and functionality of quality improvement teams may influence outcomes.

Medium-high quality quantitative studies suggest that the quality, intensity and appropriateness of quality improvement support may contribute to perceived improvement of outcomes, but not, where measured, actual improvement [42, 46, 48–51]. This may be partly explained by the number of ideas for improvement tested [49]. In other words, the more quality improvement teams perceive the approach to be relevant, credible and adequate, the more they may be willing to use the quality improvement approach, which in turn contributes to a positive perception of improvement. In relation to attributes of quality improvement teams, studies stress the importance of team stability, multi-disciplinary composition, involvement of opinion leaders and previous experience in quality improvement, but there is inconclusive evidence that these attributes are associated with better outcomes [49, 52–54]. Particularly in LMICs, alignment with existing supervisory structures may be the key to achieve a functional team [46, 48, 51, 57, 58].

Wider organisational contextual factors refer to characteristics of the organisation in which a QIC intervention is implemented, and the external system in which the facility operates [29]. Two factors emerge from the literature. Firstly, the nature of leadership has a key role in motivating health professionals to test and adopt new ideas and is crucial to develop “habits for improvement”, such as evidence-based practice, systems thinking and team problem-solving [49, 51, 54–56]. Secondly, alignment with national priorities, quality strategies, financial incentive systems or performance management targets may mobilise leadership and promote facility engagement in QIC programmes, particularly in LMIC settings [46, 48, 50, 51]; however, quality of this evidence is medium-low.

### Mechanisms of change

In relation to mechanisms of change, we identified two research questions to explore one domain each.

#### How may engagement in QICs influence health workers and the organisational context to promote better adherence to evidence-based practices?

We identified six mechanisms of change *within* an organisation (Table 4). First, participation in QIC activities may increase their commitment to change by increasing confidence in using data to make decisions and identifying clinical challenges and their potential solutions within their reach [17, 49, 51, 55, 56, 60–62]. Second, it may improve accountability by making standards explicit, thus enabling constructive challenge among health workers when these are not met [17, 62, 64–66]. A relatively high number of qualitative and mixed-methods studies of medium–high quality support these two themes. Other mechanisms, supported by fewer and lower quality studies, include improving health workers’ knowledge and problem-solving skills by providing opportunities for peer reflection [46, 48, 64, 67]; improving organisational climate by promoting teamwork, shared responsibility and bottom up discussion [60–62, 67]; strengthening a culture of joint problem solving [48, 63]; and supporting an organisational cultural shift through the development of “habits for improvement” that promote adherence to evidence-based practices [17, 56, 62].

**Table 4:** Intra-organisational mechanisms of change

Themes (No. studies)	Evidence synthesis				Quality of Evidence [ref.]	
	Description of relationship QIC component–mechanism–outcome			Contextual enablers of mechanism (or barriers)	Quantitative and mixed methods	Qualitative and review
	QIC component	Mechanism of change	Outcome			
Health professionals -knowledge, skills & problem solving (*N* = 4)	Use of continuous quality improvement approach	• Refreshed knowledge • Reinforced confidence and skills in improvement topic area • Facilitated a problem-solving approach	Change in clinical practice enabled	• Quality and appropriateness (mix of clinical and quality improvement expertise) of mentoring • Leadership and work culture open to bottom up discussion and reflection • Health workers participating in quality improvement interventions have adequate clinical competences (or a complementary clinical skills training programme is accessible)	Medium [46]	Low [48]; medium [57, 58]
Health professionals engagement, attitude and motivation (*N* = 8)	Formulating shared goals Alignment with national priorities and fit with existing practices Use of run-charts to visualise progress Dissemination of success stories Credibility of change package	• Increased motivation, by reframing improvement topic as desirable, urgent and achievable • Removed resistance to use of data • Increased Commitment to change	Increased engagement in QIC—may lead to increased success	• Intensity of mentoring to increase data literacy and use for decision-making, particularly in LMICs • Supportive leadership • Barrier: competing programmes and initiatives.	Medium [58, 59]; high [49, 57, 60]	Low [17, 51, 61]; high [57]
Organisational climate (*N* = 4)	General QIC approach	• Facilitated teamwork and multi-professional collaboration within and across departments • Facilitated bottom up dialogue and discussion		• Quality and intensity of mentoring • Wider use of improvement tools beyond unit of focus	High [60]	Low [61]; medium [62]; high [57]
Leadership (*N* = 2)	General QIC approach	• Enhanced leadership engagement • Decentralised/shared leadership promoted through encouraging bottom up problem solving	Staff morale boosted	• Previous success with quality improvement • Alignment with institutional responsibilities and participatory working culture		Low [48, 63]
Organisational structures, processes and systems (*N* = 5)	Process mapping	• Definition of standard care processes facilitated	New expectations on performance generated	• Previous success with quality improvement • Alignment with institutional responsibilities and priorities • Complementary approach (beyond QIC activities) to institutionalise new ways of working e.g. incorporation in induction or staff training; performance management frameworks for accountability at the level of health workers and/or organisation		Low [17]; medium [62, 64, 65]; high [66]
Organisational culture (*N* = 3)	General QIC approach	• Development of habits for improvement facilitated	Normalisation of new practices	• Leadership open to new practices • Health system enabling decentralised innovation		Low [17, 56]; medium [62]

The available literature highlights three key contextual enablers of these mechanisms: the appropriateness of mentoring and external support, leadership characteristics and adequacy of clinical skills. The literature suggests that external mentoring and support is appropriate if it includes a mix of clinical and non-clinical coaching, which ensures the support is acceptable and valued by teams, and if it is highly intensive, particularly in low-income settings that are relatively new to using data for decision-making and may have low data literacy [46, 48, 51, 58]. For example, in Nigeria, Osibo et al. suggests that reducing resistance to use of data for decision-making may be an intervention in itself and a pre-condition for use of quality improvement methods [58]. As for leadership characteristics, the literature stresses the role of hospital leadership in fostering a culture of performance improvement, promoting open dialogue, bottom-up problem solving, which may facilitate a collective sense of responsibility and engagement in quality improvement. Alignment with broader strategic priorities and previous success in quality improvement may further motivate leadership engagement [46, 48, 50, 51]. Adequacy of clinical skills emerges as an enabler particularly in LMICs, where implementation reports observed limited scope for problem-solving given the low competences of health workers [46] and the need for partnership with training programmes to complement clinical skills gaps [48].

#### What is it about collaboration with other hospitals that may lead to better outcomes?

This question explored inter-organisational mechanisms of change. Four themes emerged from the literature (Table 5). Firstly, collaboration may create or reinforce a community of practice, which exerts a normative pressure on hospitals to engage in quality improvement, [17, 46, 50, 63, 67–69]. Secondly, it may promote friendly competition and create *isomorphic pressures* on hospital leaders, i.e. pressure to imitate other facilities’ success because they would find it damaging not to. In reverse, sharing performance data with other hospitals offers a potential reputational gain for well-performing hospitals and for individual clinicians seeking peer recognition [17, 46, 63, 68, 69, 72] . A relatively high number of medium-high quality studies support these two themes. Thirdly, collaboration may provide a platform for capacity building by disseminating success stories and methodologies for improvement [51, 67–70]. Finally, collaboration with other hospitals may demonstrate the feasibility of improvement to both hospital leaders and health workers. This, in turn, may galvanise action within each hospital by reinforcing intra-organisational change mechanisms outlined above [51, 63, 71]. However, evidence for this comes from low-quality studies.

**Table 5:** Inter-organisational mechanisms of change

Themes (No. studies)	Evidence synthesis				Quality of Evidence [ref.]	
	Description of relationship QIC component–mechanism–outcome			Contextual enablers of mechanism (or barriers)	Quantitative and mixed methods	Qualitative and review
	QIC component	Mechanism of change	Outcome			
Shared community of practice (*N* = 7)	Collaboration with other hospitals	• Sense of community reinforced or created • Increased motivation, by supporting reframing of improvement topic as desirable, urgent and achievable	Health workers motivated and empowered to take action towards common goal	• Settings where a *community of practice* amongst clinicians exists or can be developed • Barrier: external pressures on hospitals incentivising competition v. collaboration.	Medium [46, 67–69]	Low [17, 50, 63]; medium [67, 69]
Platform for capacity building (*N* = 5)	Collaboration with other hospitals	• Platform to refine skills for improvement provided • Definition of standard care processes facilitated		• Settings with quality-focused HR systems, e.g. incorporating quality objectives in professional development and performance appraisals • Barrier: high performing hospitals have less to gain from collaboration • Barrier: Collaboration can be undermined by free-riding (not all facilities contribute equally) and social loafing (leaving it to others to support low performing hospitals)	Medium [51, 67–70]	Low [51]; medium [67, 69, 70]
Demonstration role (*N* = 3)	Collaboration with other hospitals	• Feasibility of improving outcome of focus is demonstrated	Increased engagement in QIC	• Supportive leadership • External support to disseminate success stories • Barrier: Large hospitals may have less to gain from collaboration	Medium [71]	Low [51, 63]
Friendly competition (*N* = 6)	Collaboration with other hospitals	• Reputational gain from improvement (or conversely risk of non-improvement) at individual and organisational level achieved. • Access to others’ data and benchmarking for internal gains enabled.	Normative pressures to conform (change practice and improve) created.	• Open sharing of data on mutual performance • Alignment with institutional priorities (lack of which contributes to perception that collaboration is stressful and time-consuming) • Geographically dense professional network • Non-hierarchical teams facilitating decentralised decision making • Barrier: competition for financial incentives linked to quality criteria	Medium [47, 66]	Low [17, 63]; medium [69]; high [72]

Key contextual enablers for these inter-organisational mechanisms include adequate external support to facilitate sharing of success stories in contextually appropriate ways and alignment with systemic pressures on hospital leadership. For example, a study on a Canadian QIC in intensive care units found that pressure to centralise services undermined collaboration because hospitals’ primary goal and hidden agenda for collaboration were to access information on their potential competitors [72]. The activation of isomorphic pressures also assumes that a community of practice exists or can be created. This may not necessarily be the case, particularly in LMICs where isolated working is common: a study in Malawi attributed the disappointing QIC outcomes partly to the intervention’s inability to activate friendly competition mechanisms due the weakness of clinical networks [46].

The relative benefit of collaboration was questioned in both high and low-income settings: less importance was attached to learning sessions than mentoring by participants in a study in Tanzania [57]. Hospitals may fear exposure and reputational risks [68], and high-performing hospitals may see little advantage in their participation in a collaborative [68, 72]. Hospitals may also make less effort when working collaboratively or use collaboration for self-interest as opposed to for sharing their learning [69].

**Fig. 5:**
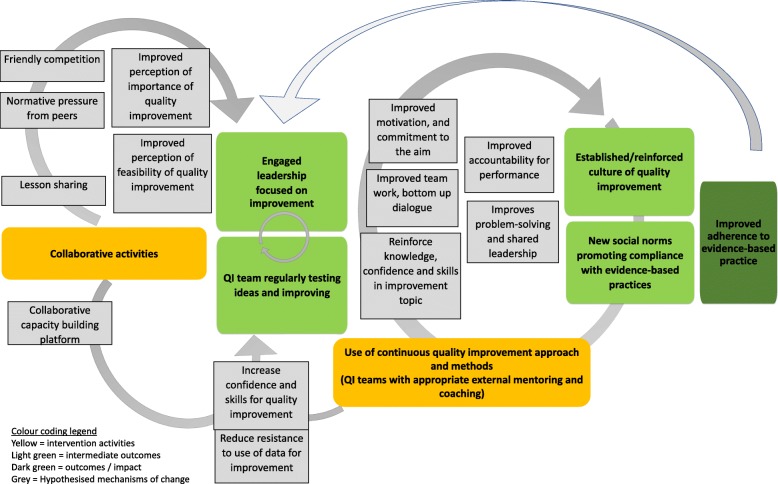
Programme theory

Figure 5 offers a visual representation of the identified intra- and inter-organisational mechanisms of change and their relationship to the intervention strategy and expected outcomes.

## Discussion

To the best of our knowledge, this is the first review to systematically explore the role of context and the mechanisms of change in QICs, which can aid their implementation design and evaluation. This is particularly important for a complex intervention, such as QICs, whose effectiveness remains to be demonstrated [6, 7, 11]. We offer an initial programme theory to understand whose behaviours ought to change, at what level, and how this might support the creation of social norms promoting adherence to evidence-based practice. Crucially, we also link intra-organisational change to the position that organisations have in a health system [33].

The growing number of publications on mechanisms of change highlights interest in the process of change. We found that participation in quality improvement collaborative activities may improve health professionals’ knowledge, problem-solving skills and attitude; teamwork; shared leadership and habits for improvement. Interaction across quality improvement teams may generate normative pressure and opportunities for capacity building and peer recognition. However, the literature generally lacks reference to any theory in the conceptualisation and description of mechanisms of change [7]. This is surprising given the clear theoretical underpinnings of the QIC approach, including normalisation process theory in relation to changes within each organisation, and diffusion of innovation theory in relation to changes arising from collaborative activities [32, 33]. We see three key opportunities to fill this theoretical gap. First, more systematic application of the Theoretical Domains Framework in design and evaluation of QICs and in future reviews. This is a synthesis of over 120 constructs from 33 behaviour change theories and is highly relevant because the emerging mechanisms of change pertain to seven of its domains: knowledge, skills, reinforcement, intentions, behaviour regulation, social influences and environmental context and resources [73, 74]. Its use would allow specification of target behaviours to change, i.e. who should do what differently, where, how and with whom, to consider the influences on those behaviours, and to prioritise targeting behaviours that are modifiable as well as central to achieving change in clinical practice [75]. Second, we recognise that emphasis on individual behaviour change theories may mask the complexity of change [76]. Organisational and social psychology offer important perspectives for theory building, for example, postulating that motivation is the product of intrinsic and extrinsic factors [77, 78], or that group norms that discourage dissent, for example, by not encouraging or not rewarding constructive criticism act as a key barrier to individual behaviour change [79]. This warrants further exploration. Third, engaging with the broader literature on learning collaboratives may also help develop the programme theory further and widen its application.

Our findings on contextual enablers complement previous reviews [16, 80]. We highlight that activating mechanisms of change may be influenced by the appropriateness of external support, leadership characteristics, quality improvement capacity and alignment with systemic pressures and incentives. This has important implications for QIC implementation. For example, for external support to be of high intensity, the balance of clinical and non-clinical support to quality improvement teams will need contextual adaptation, since different skills mixes will be acceptable and relevant in different clinical contexts. Particularly in LMICs, alignment with existing supervisory structures may be the key to achieve a functional quality improvement team [46, 48, 51, 57, 58].

Our review offers a more nuanced understanding of the role of leadership in QICs compared to previous concepts [8, 25]. We suggest that the activation of the mechanisms of change, and therefore potentially QIC success, rests on the ability to engage leaders, and therefore leadership engagement can be viewed as a key part of the QIC intervention package. In line with organisational learning theory, the leaders’ role is to facilitate a data-informed analysis of practice and act as “designers, teachers and stewards” to move closer to a shared vision [81]. This requires considerable new skills and a shift away from traditional authoritarian leadership models [81]. This may be more easily achieved where some of the “habits for improvement” already exist (13), or where organisational structures, for example, decentralised decision-making or non-hierarchical teams, allow bottom-up problem solving. Leadership engagement in QIC programmes can be developed through alignment with national priorities or quality strategies, alignment with financial incentive systems or facility performance management targets, particularly as external pressures may compete with QIC aims. Therefore, QICs design and evaluation would benefit from situating these interventions in the health system in which they occur.

Improving skills and competencies in using quality improvement methods is integral to the implementation of QIC interventions; however, the analysis of contextual factors suggests that efforts to strengthen quality improvement capacity may need to consider other factors as well as the following: firstly, the availability and usability of health information systems. Secondly, health workers’ data literacy, i.e. their confidence, skills and attitudes towards the use of data for decision-making. Thirdly, adequacy of health workers’ clinical competences. Fourth, leaders’ attitudes to team problem solving and open debate, particularly in settings where organisational culture may be a barrier to individual reflection and initiative. The specific contextual challenges emerging from studies from LMICs, such as low staffing levels and low competence of health workers, poor data systems, and lack of leadership echo findings on the limitations of quality improvement approaches at facility-level in resource constrained health systems [1, 82]. These may explain why group-problem solving strategies, including QICs, may be more effective in moderate-resource than in low-resource settings, and their effect larger when combined with training [11]. The analysis on the role of context in activating mechanisms for change suggests the need for more explicit assumptions about context-mechanism-outcome relationships in QIC intervention design and evaluation [15, 83]. Further analysis is needed to determine whether certain contextual factors related to capacity should be a precondition to justify the QIC approach (an “investment viability threshold”) [84], and what aspects of quality improvement capacity a QIC intervention can realistically modify in the relatively short implementation timeframes available.

While we do not suggest that our programme theory is relevant to all QIC interventions, in realist terms, this may be generalizable at the level of theory [18, 20] offering context-mechanism-outcome hypotheses that can inform QIC design and be tested through rigorous evaluations, for example, through realist trials [85, 86]. In particular, there is a need for quantitative analysis of hypothesised mechanisms of change of QICs, since the available evidence is primarily from qualitative or cross-sectional designs.

Our review balances principles of systematic reviews, including a comprehensive literature search, double abstraction, and quality appraisal, with the reflective realist review approach [19]. The realist-inspired search methodology allowed us to identify a higher number of papers compared to a previous review with similar inclusion criteria [16] through active search of qualitative studies and grey literature and inclusion of low quality literature that would have otherwise been excluded [41]. This also allowed us to interrogate what did *not* work, as much as what did work [19, 22]. By reviewing literature with a wide range of designs against a preliminary conceptual framework, by including literature spanning both high- and low-resource settings and by exploring dissonant experiences, we contribute to understanding QICs as “disruptive events within systems” [87].

Our review may have missed some papers, particularly because QIC programme descriptions are often limited [7]; however, we used a stringent QIC definition aligned with previous reviews, and we are confident that thematic saturation was achieved with the available studies. We encountered a challenge in categorising data as “context” or “mechanism”. This is not unique and was anticipated [88]. Double review of papers in our research team minimised subjectivity of interpretation and allowed a deep reflection on the role of the factors that appeared under both dimensions.

## Conclusion

We found some evidence that appropriateness of external support, functionality of quality improvement teams, leadership characteristics and alignment with national systems and priorities may influence QIC outcomes, but the strength and quality of the evidence is weak. We explored how QIC outcomes may be generated and found that health professionals’ participation in QIC activities may improve their knowledge, problem-solving skills and attitude; team work; shared leadership and the development of habits for improvement. Interaction across quality improvement teams may generate normative pressure and opportunities for capacity building and peer recognition. Activation of mechanisms of change may be influenced by the appropriateness of external support, leadership characteristics, the adequacy of clinical skills and alignment with systemic pressure and incentives.

There is a need for explicit assumptions about context-mechanism-outcome relationships in QIC design and evaluation. Our review offers an initial programme theory to aid this. Further research should explore whether certain contextual factors related to capacity should be a precondition to justify the QIC approach, test the emerging programme theory through empirical studies and refine it through greater use of individual behaviour change and organisational theory in intervention design and evaluation.

## Supplementary information

**Additional file 1.** Search terms used.

**Additional file 2.** Systematic review alignment with RAMESES publication standards checklist.

**Additional file 3.** Quality appraisal of included studies.

## Abbreviations

IQR: Inter-quartile range
LMIC: Low and middle-income country
MES: Median effect size
MUSIQ: Model for understanding success in improving quality
QIC: Quality improvement collaborative
STROBE: Strengthening the reporting of observational studies in epidemiology
