# Supplementary material for: How and under what circumstances do quality improvement collaboratives lead to better outcomes? A systematic review
Source: Implement Sci. 2020 May 4;15:27. doi: 10.1186/s13012-020-0978-z (PMC7199331; doi:10.1186/s13012-020-0978-z)
Supplement: Supplementary file 1 — Additional file 1. Search terms used. [file 13012_2020_978_MOESM1_ESM.docx]

**Additional file 1: Search terms used**

**Database: Medline**

**Concept 1: Quality improvement collaborative**

1 "learning collaborative*" (220)

2 "improvement collaborative (335)

3 "performance improvement collaborative*".mp. (3)

4 "quality improvement collaborative*".mp. (270)

5 "quality collaborative*".mp. (167)

6 (Breakthrough adj (series or approach or network or project*)).mp. (142)

7 "collaborative network".mp. (313)

8 "improvement network".mp. (580)

9 (quality improv* adj5 collaborat*).mp. (601)

10 "model for improvement".mp. (337)

11 "collaborative improvement".mp. (59)

12 "Breakthrough collaborative*".mp. (32)

13 michigan intensive care unit.mp. (3) - NOTE: I have included this to get the Michigan theory of change paper which did not appear in any other search

14 1 or 2 or 3 or 4 or 5 or 6 or 7 or 8 or 9 or 10 or 11 or 12 or 13 (2355)

**Filters (for quality improvement and process evaluation)**

15 Qualitative Research/ (37744)

16 Health Services Research/og [Organization & Administration] (2634)

17 Quality Assurance, Health Care/ or Quality Improvement/ or Total Quality Management/ (83330)

18 Program Evaluation/ (58333)

19 Interprofessional Relations/ (49467)

20 "Surveys and Questionnaires"/ or "Attitude of Health Personnel"/ (493019) – these were subfilters of the Mixed Method study filter.

**Filtered concept:**

21 14 and (15 or 16 or 17 or 18 or 19 or 20) (921)

**Database: PubMED**

**((((((("quality improvement collaborative"[Title/Abstract]) OR "quality collaborative"[Title/Abstract]) OR "improvement collaborative"[Title/Abstract]) OR "learning collaborative"[Title/Abstract]) OR "Breakthrough series"[Title/Abstract]) OR "Breakthrough collaborative"[Title/Abstract]) OR "collaborative network"[Title/Abstract]) OR "quality improvement network"[Title/Abstract] AND Evaluation Studies[ptyp]**

Quality improvement collaborative

Quality collaborative

Improvement collaborative

Learning collaborative

Breakthrough series

Breakthrough collaborative

Collaborative network

Quality improvement network

Learning collaborative

Filtered by: Evaluation studies.

**Database: CINHAL (EBSCOhost)**

**Concept: quality improvement collaborative**

learning collaborative

improvement collaborative

performance improvement collaborative

quality improvement collaborative

quality collaborative

Breakthrough series

Breakthrough collaborative

Collaborative network

model for improvement

Collaborative improvement

Collaborative learning

Michigan intensive care unit

**Filters:**

"Process Assessment (Health Care)" OR "Summative Evaluation Research" OR "Formative Evaluation Research") OR Quality Improvement OR "Quality Management, Organizational" OR "Evaluation and Quality Improvement Program"

**Database: EMBASE**

**Concept 1: Quality improvement collaborative**

1 "learning collaborative*"

2 "improvement collaborative

3 "performance improvement collaborative*".

4 "quality improvement collaborative*"

5 "quality collaborative*"

6 (Breakthrough adj (series or approach or network or project*))

7 "collaborative network"

8 "quality improvement network”

9 (quality improv* adj5 collaborat*)

10 "collaborative improvement"

11 "Breakthrough collaborative*"

12 michigan intensive care unit.mp. (3) - NOTE: I have included this to get the Michigan theory of change paper which did not appear in any other search

13 1 or 2 or 3 or 4 or 5 or 6 or 7 or 8 or 9 or 10 or 11 or 12

**Filters (for quality improvement and process evaluation)**

14 Total quality management

15 Program Evaluation OR evaluation OR “organisation and management”

16 Qualitative research

17 Health personnel attitude

18 15 OR 16 OR 17

19 18 AND 14

20 13 AND 19

**Database: Global Health**

1 "learning collaborative*"

2 "improvement collaborative

3 "performance improvement collaborative*".

4 "quality improvement collaborative*"

5 "quality collaborative*"

6 (Breakthrough adj (series or approach or network or project*))

7 "collaborative network"

8 "quality improvement network”

9 (quality improv* adj5 collaborat*)

10 "collaborative improvement"

11 "Breakthrough collaborative*"

12 michigan intensive care unit.mp. (3) - NOTE: I have included this to get the Michigan theory of change paper which did not appear in any other search

13 “Model for improvement”

14 1 or 2 or 3 or 4 or 5 or 6 or 7 or 8 or 9 or 10 or 11 or 12 or 13

**Filters (for quality improvement and process evaluation)**

15 (Qualitative analysis or health services or health care workers)

16 (quality controls or health care)

17 Program Evaluation or innovation adoption

18 health care workers

19 14 and (15 or 16 or 17 or 18)

**Grey literature: USAID ASSIST website**

Improvement science AND

Collaborative improvement AND

Report (15) OR

Short report (3) OR

Case study (1) OR

Journal article (2)
