# Supplementary material for: How and under what circumstances do quality improvement collaboratives lead to better outcomes? A systematic review
Source: Implement Sci. 2020 May 4;15:27. doi: 10.1186/s13012-020-0978-z (PMC7199331; doi:10.1186/s13012-020-0978-z)
Supplement: Supplementary file 2 — Additional file 2. Systematic review alignment with RAMESES publication standards checklist. [file 13012_2020_978_MOESM2_ESM.docx]

**Additional file 2 – Systematic review alignment with RAMESES publication standards checklist**

| **#** | **Checklist item** | **Reported** |
| --- | --- | --- |
| 1 | Title identified as realist review | No – this is because this is a realist-inspired review. We have followed the realist review methodology but noted and justified some points of departure from it. |
| 2 | Abstracts should ideally contain brief details of the study’s background, review question or objectives; search strategy; methods of selection, appraisal, analysis and synthesis of sources; main results; and implications for practice. | Yes – p. 1 |
| 3 | Explain why the review is needed and what it is likely to contribute to existing understanding of the topic area. | yes – Background |
| 4 | State the objective(s) of the review and/or the review question(s). Define and provide a rationale for the focus  of the review. | yes – p. 5 (Background) |
| 5 | Any changes made to the review that was initially planned should be briefly described and justified. | Not relevant |
| 6 | Explain why realist synthesis was considered the most appropriate method to use. | Yes – p. 5 (Methods) |
| 7 | Describe and justify the initial process of exploratory scoping of the literature. | Yes – p. 5-6 |
| 8 | State and provide a rationale for  how the iterative searching was done. Provide details on all the sources accessed for information in the synthesis. If individuals familiar with the relevant literature and/or topic area were contacted, indicate how they were identified and selected. | Yes - p. 8. However, involvement of individuals familiar with the literature was more limited than we would have wanted it to be, and this is acknowledged as a limitation – p. 23 |
| 9 | Explain how judgements were made about including and excluding data from documents, and justify these. | Yes – p. 8 |
| 10 | Describe and explain which data or information were extracted from the included documents and justify this selection. | Yes – p. 9 |
| 11 | Describe the analysis and synthesis processes in detail. This section should include information on the constructs analyzed and describe the analytic process. | Yes – p. 10 |
| 12 | Provide details on the number of documents assessed for eligibility and included in the review with reasons for exclusion at each stage, as well as an indication of their source of origin (for example, from searching databases, reference lists and so on). | Yes – p. 10 and Figure 3 |
| 13 | Provide information on the characteristics of the documents included in the synthesis. | Yes – p. 10 – 11 and Table 1 |
| 14 | Present the key findings with a specific focus on theory building and testing. | Yes – p. 11 – 18 and Figure 1 and 4 |
| 15 | Summarize the main findings, taking into account the synthesis’ objective(s), research question(s), focus and intended audience(s). | Yes – p. 18 (Discussion, first paragraph) |
| 16 | Discuss both the strengths of the review and its limitations. | Yes – p. 22-23 |
| 17 | Where applicable, compare and contrast the synthesis’ findings with the existing literature (for example, other  reviews) on the same topic. | Yes – p. 18 - 22 |
| 18 | List the main implications of the findings and place these in the context of other relevant literature. If appropriate, offer recommendations for policy and practice. | Yes – p. 18 -24 |
| 19 | Provide details of funding source (if any) for the synthesis, the role played by the funder (if any) and any conflicts of interests of the reviewers. | Yes – end of manuscript |
