# Supplementary material for: How and under what circumstances do quality improvement collaboratives lead to better outcomes? A systematic review
Source: Implement Sci. 2020 May 4;15:27. doi: 10.1186/s13012-020-0978-z (PMC7199331; doi:10.1186/s13012-020-0978-z)
Supplement: Supplementary file 3 — Additional file 3. Quality appraisal of included studies. [file 13012_2020_978_MOESM3_ESM.docx]

**Appendix 3 – Quality appraisal of included studies**

| **Study no** | **Author** | **Score** | **Quality rating** | **Potential bias and key limitations** |
| --- | --- | --- | --- | --- |
| **Study type: Qualitative. Checklist used : CASP. Max score: 10** | | | | |
| 2 | Ament S et al. | 7/10 | Medium | Single case study design which does not study deviant cases.  Selection bias: Most of the respondents were the coordinators or key implementation people from hospitals that have achieved successful implementation. |
| 3 | Baker U. et al. | 9/10 | High | Independent evaluation from implementation and sampling is based on facility level functionality.  Response/Social desirability bias: The health workers might have given a favourable response to report better improvements.  Selection bias: Perspective of only one group of potential respondents’ i.e. only health workers’ perspective taken. |
| 8 | Carter P. et al. | 7/10 | Medium | Recall bias: Interviews undertaken at the end only, not as quality improvement was in progress, so recall bias may have been an issue, and also more time for participants to analyse their experience.  Selection bias: Sampling limited to available respondents. |
| 11 | Dainty K. et al. | 9/10 | High | Strong purposive sampling and analysis  Attrition bias: The ICUs that did not respond may have had a different experience. |
| 15 | Duckers et al. | 7/10 | Medium | Selection bias: Self-selected participants from the first wave of programme i.e. the early adopters and enthusiasts with previous QI experience. |
| 19 | Jaribu J. et al | 7/10 | Medium | Study does not discuss deviant cases or data.  Social desirability bias/Response bias: Possibility of respondents stating successes and positive results more as the project implementer were the interviewers.  Selection bias: Respondents were active participants in QI. View of pregnant women or community  Members and health workers who did not participate not taken.  Recall bias: The evaluation was carried out more than a year later than the project activities |
| 27 | Parand A. et.al. | 9/10 | High | Lacks objective data to test if any of the strategies actually did deliver more sustained results. |
| 32 | Stone et. al | 7/10 | Medium | Study does not discuss deviant cases or data.  Inadequate justification of single group interview in each participating hospital v. individual interviews. More nuanced perspective may have emerged. |
| **Study type: Cross-sectional. Checklist used: STROBE. Max score: 22** | | | | |
| 13 | Duckers et al. | 18/22 | High | Nonresponse bias: Low response rate as only  33% of the targeted population completely filled out the questionnaire.  Measurement bias: No pretesting of validity of survey instrument could have lead of measurement bias.  Response Bias: Survey is based on self-reports |
| 14 | Duckers et al. | 18/22 | High | Response Bias: Data outcomes are self-reported; therefore higher possibility of social desirability.  Measurement bias: The model used didn’t consider the variation that may exist in different QI interventions e.g. complexity and time consumed. |
| 16 | Duckers et al. | 17/22 | Medium | Nonresponse bias: Low response rate of 57%, high chances of possibly positively skewed results.  Expectation bias: Positive conclusions based on self-reporting and unsupported by design seem to be based on perceived success by few individuals. |
| 20 | Linnander, E. et. al. | 16/22 | Medium | Nonresponse bias: Low response rate of 57%, high chances of possibly positively skewed results as the ones who remained in sample were more likely to be engaged and improved.  Response bias: Based on self-reported patient satisfaction scores. |
| 25 | Nembhard, I.M | 17/22 | Medium | Nonresponse bias: Response rate is 68% but comparable to other studies of quality improvement collaborative.  Possible selection bias: Study teams that had more positive views might have been selected.  Response bias: Lacks objective outcome data and uses self-reported measures of participation.  Evaluation bias: Possibility of evaluator bias given that only participants under Institute for Healthcare improvement collaborative were studied. |
| 28 | Pinto A. et al | 17/22 | Medium | Nonresponse bias: Response rate is 52%.  Response bias: Self-reported un-validated measures on perceived context, effectiveness and efficacy of programme.  this study relates  Generalisability: Limited generalisability of the results as the study was focused only on one specific collaborative programme |
| 30 | Schouten et al. | 17/22 | Medium | Response bias: Self-reported data.  Selection bias: Comparison between hospitals volunteering to participate and all other hospitals.  Reporting bias: Model does not take into account severity of stroke, patient factors affecting outcome. |
| **Study type: Uncontrolled before-after. Checklist used STROBE. Max score: 22** | | | | |
| 1 | Amarasingham, R., et al. | 18/22 | High | Selection bias: Study uses uncontrolled study design and ICUs that participated, which may respond differently to the ones who didn’t participate.  Response bias: Independent variable data is based on self-reports. |
| 5 | Benn, J. et. al. | 19/22 | High | Selection bias: Study uses uncontrolled study design.  Response bias: Independent variable and outcome data is based on self-reports.  Measurement bias: The order of entry of variables in regression model is not theoretically justified.  Recall bias: Some items were about the conditions present 15 months prior to the survey |
| 23 | Mills and Weeks | 12/22 | Medium | Selection bias: Study uses uncontrolled study design  Response bias: Independent variable data is based on self-reports.  Limited details on response rate, measures assessed, administration of survey. Acknowledgement of limitations but conclusions not adequately cautious. |
| **Study type: Non-randomised controlled trial. Checklist used : STROBE. Max score: 22** | | | | |
| 7 | Carlhed, R. et. al. | 19/22 | High | Selection bias: The hospitals in the intervention were the ones who volunteered to participate, whereas the control hospitals were selected from the hospitals that did not accept the invitation.  Discussion suggests a relationship between context and outcome but data analysis and methods not described. |
| 22 | McInnes, D. K., et al. | 19/22 | High | Response bias: Self-reported outcome measures such as culture, teamwork.  Measurement bias: The clinical scores used in the study have relatively low reliability. |
| **Study type: Mixed-methods. Checklist used : Mixed Methods Appraisal Tool. Max score: 15** | | | | |
| 4 | Benn, J. et. Al | 13/15 | High | Generalisability: Small sample is studied.  Difficult to ascertain bias as the article does not allow the study of hypothesis rather formulating one. |
| 6 | Burnett et al | 9/15 | Medium | Selection bias: Homogeneous group with high self-reported readiness are study respondents and no frontline worker surveyed. |
| 9 | Colbourn et. Al | 8/15 | Medium | Difficult to ascertain bias as cross-sectional design does not allow the study of hypothesis and survey data seems to be incomplete for hypothesis testing. |
| 24 | Nembhard, I.M | 9/15 | Medium | Nonresponse bias: Response rate is 68% but comparable to other studies of quality improvement collaborative.  Selection bias: Study teams that had more positive views might have been selected.  Response bias: Lacks objective outcome data and uses self-reported measures of participation.  Evaluation bias: Possibility of evaluator bias given reliance of single evaluator from Institute for Healthcare improvement. |
| 26 | Osibo B. et al. | 12/15 | Medium | Response/Social desirability bias: The respondents might have given a favourable response to report better improvements. |
|  |  |  |  |  |
| **Study type: External reviews or case descriptions. Checklist used : CASP Max score: 10** | | | | |
| 10 | Cstambas TT et al. | 3/10 | Low | Evaluation Bias: This is a non-peer reviewed report to donor. Reports of improvements in compliance with practices not presented with adequate reflection on limitations of design, data and effect. |
| 12 | Dixon Woods et al | 4/10 | Low | The article is highly useful for formulating hypothesis; describes a theory developed by implementers and social scientists. Difficult to ascertain and there is lack of methods, data and analysis. |
| 17 | Feldman-Winter L. et al. | 1/10 | Low | Generalisability: Describes a single case study, transferability is limited.  Method of data collection and analysis are not clear.  Evaluation bias: Possibility of evaluator bias as the narratives is summarized by organisers itself. |
| 18 | Horbar J. et al. | 4/10 | Low | Response bias: Has a weak methodology; results based only on implementers' experience. |
| 21 | Marquez L. et al. | 1/10 | Low | Response bias: Has a weak methodology; results based only on implementers' experience and various heavily biased studies. |
| 29 | Rahimzai M. et al | 4/10 | Low | Selection bias: Only few facilities were part of intervention with a high possibility of being the most promising ones. Only one case study presented.  Evaluation bias: Reflections based on implementers' perception only and not participants. |
| 31 | Sodzi-Tettey et al. | 2/10 | Low | Evaluation bias: Possibility of evaluator bias as the reflections are based on senior leadership from implementers, health services offices and partners and not health workers. Narratives are summarized by implementers itself. There is lack of evidence against arguments given. |
